# The Identification of Pathway Markers in Intracranial Aneurysm Using Genome-Wide Association Data from Two Different Populations

**DOI:** 10.1371/journal.pone.0057022

**Published:** 2013-03-06

**Authors:** Burcu Bakir-Gungor, Osman Ugur Sezerman

**Affiliations:** 1 Department of Genetics and Bioinformatics, Faculty of Arts and Sciences, Bahcesehir University, Istanbul, Turkey; 2 Advanced Genomics and Bioinformatics Research Center, UEKAE, TUBITAK, Kocaeli, Turkey; 3 Biological Sciences and Bioengineering, Faculty of Engineering and Natural Sciences, Sabancı University, Istanbul, Turkey; National Taiwan University, Taiwan

## Abstract

The identification of significant individual factors causing complex diseases is challenging in genome-wide association studies (GWAS) since each factor has only a modest effect on the disease development mechanism. In this study, we hypothesize that the biological pathways that are targeted by these individual factors show higher conservation within and across populations. To test this hypothesis, we searched for the disease related pathways on two intracranial aneurysm GWAS in European and Japanese case–control cohorts. Even though there were a few significantly conserved SNPs within and between populations, seven of the top ten affected pathways were found significant in both populations. The probability of random occurrence of such an event is 2.44E−36. We therefore claim that even though each individual has a unique combination of factors involved in the mechanism of disease development, most targeted pathways that need to be altered by these factors are, for the most part, the same. These pathways can serve as disease markers. Individuals, for example, can be scanned for factors affecting the genes in marker pathways. Hence, individual factors of disease development can be determined; and this knowledge can be exploited for drug development and personalized therapeutic applications. Here, we discuss the potential avenues of pathway markers in medicine and their translation to preventive and individualized health care.

## Introduction

Many examples of genome-wide association studies (GWAS) have pointed out the scarcity of the many possible variants that can contribute to the explanation of a small percentage of the estimated heritability for complex diseases. Identifying marker single nucleotide polymorphisms (SNPs) specific to a complex disease or developing genetic risk prediction tests [1]–[10] thus constitutes a major challenge. Multiple factors (e.g. SNPs, miRNAs, metabolic and epigenetic factors) may target different sets of genes in the same pathway, thus affecting the pathway’s function. In contrast to isolated molecules, network and pathway-oriented analyses are thought to capture pathological perturbations and hence, better explain predisposition to disease [11]. The alterations in the functionality of common, disease specific combinations of pathways may be the universal cause of the disease development mechanism. This realization suggests that therapeutics of the future may possibly be created while keeping cellular networks and biological pathways in mind [12]. In this regard, pathway based approaches to GWAS search for multiple genes in the same biological pathway, where the common variations in each of these genes have little effect on disease risk [13]–[21]. The potential of GWAS on disparate populations to uncover the links between genetics and pathogenesis of human complex diseases has been discussed in the literature [22]. One reason cited is the risk variants can vary in their occurrence across populations [23], [24]. For example, while the high-risk variant at MYBPC3 gene is observed with a frequency of ∼4% in cardiomyopathy patients in Indian populations; this variant is rare or absent in other populations [25]. Another reason is the difference in allele frequencies and biological adaptations among populations, which in turn affects the detectability and importance of risk variants. The identification of a variant might be easier in some populations when compared to that of other populations since the particular histories of recombinations, mutations, and divergences of genealogical lineages in the various populations affect the mappability of a variant. This situation is observed in the variants of TCF7L2 and KCNQ1 genes in type 2 diabetes [26], [27]. Also, a review paper by Stranger *et al.* has been pointed out that studying additional populations in GWAS may provide valuable insights for current and future research in medical genetics [28]. Inspired by these research efforts, in this study, we hypothesize that the few SNPs that are identified in GWAS and their associated genes may be targeting the same pathway combinations, and these biological pathways show higher conservation across populations. If the combination of these pathways does not function properly, a specific disease may develop. Therefore, affected pathways may be conserved across populations, making them potential markers for multifactorial diseases. Until now, no SNP or gene markers have been identified for complex diseases, that have high explanatory power and that can be applied across populations. We propose pathway markers, as an alternative that shows more conservation across populations. That’s why these markers can even be used at individual level, enlightening individual disease development mechanisms.

Intracranial aneurysm (IA, OMIM 105800) is a cerebrovascular disease that affects approximately one individual out of fifty [29]. IA is thought to be a major public health concern since the rupture of an IA leads to subarachnoid hemorrhage (SAH), which is a destructive subset of stroke [30]. One third of the patients with SAH die within the initial weeks after the bleed and the rest end up with severe physical disabilities [31]. Both environmental risk factors such as smoking, hypertension, excessive alcohol intake; and non-modifiable risk factors such as family history of IA, female gender and systemic diseases (e.g. polycystic kidney disease and vasculr type of Ehlers Danlos disease) are accepted to have a role in the development of IA and SAH [32]–[38]. Since the subjects with familial preponderance of IA have a higher risk of IA, genetic components are thought to correlate with the tendency of developing IA. Four recent GWAS identified some variants associated with IA [39]–[42]. In JP population, five SNPs (rs1930095 (P = 1.31×10–5), rs4628172 (P = 1.32×10–5), rs7781293 (P = 2.78×10–5), rs7550260 (P = 4.93×10–5), rs9864101 (P = 3.63×10–5)) were associated with IA [40], [42]. In EU population, five loci were found to be strongly related with IA; chromosomes 18q11.2 (rs11661542, OR = 1.22, P = 1.1×10−12), 10q24.32 (rs12413409, OR = 1.29, P = 1.2×10−9), 13q13.1 (rs9315204, OR = 1.20, P = 2.5×10−9), 8q11.23–q12.1 (rs10958409, rs9298506, OR = 1.28, P = 1.3×10−12), 9p21.3 (rs1333040, OR = 1.31, P = 1.5×10−22) [39] and a further 14 loci displayed suggestive association [43]. However, these variants explain only a small percentage of the familial risk of IA, and this situation makes genetic risk prediction tests currently unfeasible for IA [31]. Previously, we developed PANOGA (Pathway and Network Oriented GWAS Analysis), a novel methodology to devise functionally important KEGG pathways through the identification of SNP targeted genes within these pathways [44]–[46]. PANOGA combines nominally significant evidence of genetic association with current knowledge of protein-protein interaction (PPI) networks and functional information of selected SNPs. Here, we applied PANOGA on two IA GWAS separately: i) Finnish, Dutch (European, EU) population of 1701 cases and 7409 control cohorts [39], [41], ii) Japanese (JP) population of 1069 cases and 904 controls [40]. Even though there were not so many common disease predisposing SNPs and commonly targeted genes between these two populations, the identification of 7 common pathways in the top 10 pathways demonstrated the relevance of our pathway-oriented approach. In the following sections, we will discuss our findings.

## Results

In our analysis, we have included 44,351 SNPs from the EU population specific dataset, and 14,034 SNPs from the JP population specific dataset with p-values <0.05, where the genotypic p-value of a SNP is calculated via Cochran-Armitage trend test. Only 576 of these SNPs were common between the two populations. To identify the biological pathways with the genes responsible for IA susceptibility, for each dataset, we applied the affected SNP functionalization, SNP to gene mapping, gene-wise weighted p-value calculation, sub-network identification and functional enrichment steps of PANOGA. The details of these steps are explained in the Materials and Methods section.

All available human KEGG pathways, 246 in total, were tested for their possible role in IA development mechanism. Among these pathways, 103 pathways were detected in EU population (as shown in Table S1) and 102 pathways were detected in JP population (as shown in Table S2) with corrected p-values less than E−4. 91 of these pathways were commonly found in both populations (as shown in Table S3). Next, we calculated the rankings of each identified pathway in each population and found that the correlation between the two studies was significant (Spearman’s r2 = 0.71, P<10–6). Pairwise correlation of pathway statistics between two studies (which were carried out on independent populations with different ethnicities) should indicate common genetic variation associated with IA. As shown in Table 1, 12 of the top 20 (P = 4.09E−60) and 7 of the top 10 (P = 2.44E−36) affected pathways were commonly identified in both EU and JP populations. In these 12 commonly identified pathways, while 95 and 81 genes are uniquely targeted by disease predisposing SNPs in EU and JP populations respectively, only 25 genes (as shown in Table S4) are targeted by SNPs in both populations. In the 7 commonly identified pathways, while 15 of the SNP targeted genes (STGs, shown in Table S4) are common to both populations. 62 and 51 of the STGs are unique to EU and JP populations, respectively. In these 7 commonly found pathways, there were 724 and 195 SNPs unique to EU and JP populations, respectively, as well as 6 SNPs which were common. There were very few commonly affected SNPs/genes and many distinct sets of SNPs/genes targeting the same pathways for each population. This finding strongly supports our hypothesis. Hence, if one follows a gene or SNP oriented approach, crucial information for disease development mechanism might be missed. Instead, we emphasize here the importance of a pathway-oriented approach to investigate the etiology of IA. As shown in Table 1, these 7 pathways in the top 10 are MAPK signaling, Cell cycle, TGF-beta signaling, Focal adhesion, Adherens junction, Regulation of actin cytoskeleton, and Neurotrophin signaling pathways. For each commonly found pathway, we checked the numbers of STGs, typed SNPs, separately for EU and JP populations, and the commonality of these entities between the two populations. For example, in the MAPK signaling pathway, there were 14 and 18 STGs in EU and JP populations, respectively. Among these genes, 2 (MAP3K7, NFATC2, as shown in Table S4) were common, indicating that the same pathways can be targeted via independent genes in diverse populations. There were 133 and 43 typed SNPs in EU and JP populations, respectively, and among these SNPs, only 1 (rs791062) was common. In addition to these typed SNPs that were commonly identified in both populations, the commonly identified SNP targeted genes harbor other disease predisposing SNPs in different populations. For example, the MAP3K7 gene is associated with 28 other typed SNPs found in the EU population but not in the JP population. These observations were true for all the 7 commonly found pathways and the genes within them. These results show the relevance of our pathway-oriented approach and indicate that if there is a problem in these seven pathways, the disease is more likely to occur.

**Table 1:** The top 20 over-represented pathways, identified for both EU and JP populations.

	P-values		Rank		# of Associated SNPs in GWAS		# of Common SNPs in GWAS	# of SNP Targeted Genes (STGs)		# of Common STGs	% Common Genes in Both Populations		Common SNPs in GWAS
KEGG Term	EU	JP	EU	JP	EU	JP	EU∩JP	EU	JP	EU∩JP	EU	JP	EU∩JP
**MAPK signaling pathway**	3.53E−27	2.70E−18	1	8	133	43	11	14	18	2	14.29	11.11	rs791062
**Cell cycle**	2.35E−25	2.81E−19	2	4	76	18	1	11	10	2	18.18	20	rs744910
**TGF-beta signaling pathway**	6.26E−24	2.41E−17	3	9	126	20	3	15	9	5	33.33	55.56	rs2053423,rs1440375, rs744910
*ErbB signaling pathway*	9.52E−22	2.47E−15	4	16	50	15	0	6	4	0	0	0	
**Focal adhesion**	9.55E−22	5.60E−21	5	2	117	45	1	21	14	5	23.81	35.71	rs4678167
Proteasome	2.36E−21	4.55E−11	6	35	32	1	0	6	1	0	0	0	
**Adherens junction**	4.91E−19	2.58E−21	7	1	85	34	1	13	11	2	15.38	18.18	rs1561798
Notch signaling pathway	2.14E−18	4.74E−12	8	31	26	13	0	8	4	1	12.5	25	
**Regulation of actin cytoskeleton**	2.28E−18	4.05E−17	9	10	102	36	11	18	14	1	5.556	7.143	rs4678167
**Neurotrophin signaling pathway**	2.49E−18	1.93E−18	10	7	68	14	00	7	7	1	14.29	14.29	
Chronic myeloid leukemia	2.62E−18	8.13E−11	11	36	54	12	1	4	4	1	25	25	rs744910
Apoptosis	7.37E−18	1.71E−8	12	58	17	10	0	8	6	1	12.5	16.67	
*Pathways in cancer*	1.16E−17	9.38E−19	13	6	147	48	1	16	19	2	12.5	10.53	rs744910
Tight junction	1.84E−17	4.68E−14	14	21	98	37	4	14	11	6	42.86	54.55	rs4578183,rs4654383, rs955749, rs2276266
Long-term potentiation	2.25E−17	2.21E−13	15	24	140	23	0	13	10	3	23.08	30	
Measles	1.06E−16	3.42E−7	16	72	94	16	0	8	6	0	0	0	
*T cell receptor signaling pathway*	1.62E−16	1.97E−15	17	15	63	28	0	7	13	0	0	0	
*Nucleotide excision repair*	3.66E−16	8.84E−15	18	18	70	12	0	6	7	1	16.67	14.29	
Chemokine signaling pathway	1.15E−15	8.17E−12	19	32	193	26	0	13	13	2	15.38	15.38	
*Calcium signaling pathway*	3.27E−15	1.37E−15	20	12	123	42	11	21	16	8	38.1	50	rs7298821

7 commonly identified out of the top 10 affected pathways in both populations are shown in bold. 12 commonly identified pathways out of the top 20 are shown in italic.

We also wanted to check the validity of our findings (the comparison of our significant pathway list with known IA related pathways). Since there is no such gold standard dataset, we compared with IA related pathways in KEGG Disease Pathways Database using “aneurysm” as a keyword. This search returned 3 disease terms and 12 pathways as associated with these disease terms. These disease terms were: i) H00801, “Familial thoracic aortic aneurysm and dissection (TAAD) and Aortic aneurysm familial thoracic type (AAT)”; ii) H00800, “Loeys-Dietz syndrome (LDS), which is characterized by arterial aneurysms”; and iii) H00579, “Hereditary angiopathy with nephropathy, aneurysms, and muscle cramps (HANAC)”. 7 of these 12 IA related pathways were also found in our analysis in the top 20 pathway list. These 7 identified pathways were MAPK signaling, TGF-beta signaling, Calcium signaling, Focal adhesion, Adherens junction, Tight junction, Regulation of actin cytoskeleton.

Next, we searched for the affected pathways using the gene expression data obtained from ruptured and unruptured IA patients of Japanese ethnicity as cases as well as from arteriovenous malformation feeders with Japanese ethnicity as intracranial controls [47]. Even though gene expression and GWAS data do not derive from the same samples, the enriched pathways might show commonalities. Therefore, we mapped the differentially expressed genes to PPI and proceeded with the following steps of PANOGA to detect affected pathways. The top 20 over-represented KEGG pathways identified for gene expression data are shown in Table S5. No significant correlation found between the rankings of the affected pathways, obtained from GWAS and expression data (Spearman’s r2 = 0.225). The transcriptomics data only includes genes with significant changes in expression levels. Whereas, the effect of a SNP, which is found to be significant in GWAS, could be observed at different levels, e.g., splice variants, mutant protein products, post-translational modifications, a possible change in protein folding, a possible change in the interaction partner of a protein. Compared to the top ten pathways identified GWAS in EU and JP populations, the Ribosome pathway is also found by GWAS data on the Japanese population (with 5th ranking); ErbB signaling pathway and Proteasome pathways are also found by GWAS data on European population (with 4th and 6th rankings, respectively); Adherens Junction (AJ), Focal Adhesion (FA) and Neurotrophin Signaling (NS) pathways are also found by GWAS data on both Japanese and European populations (with 1st and 7th (AJ), 5th and 2nd (FA), 7th and 10th rankings (NS), respectively). In these 6 pathways (Adherens junction, Focal adhesion, ErbB signaling, Neurotrophin signaling, Ribosome, Proteasome pathways), 25 out of 379 genes were commonly identified with GWAS results. Among these genes, PTPRB gene, as part of the Adherens Junction pathway, is known to have a crucial role in blood vessel remodeling and angiogenesis. Even though this gene is not found to be differentially expressed in the Japanese population, rs1561798 variant, which is found in this gene, is found to be significant in the GWAS of both European and Japanese populations. Interestingly, another gene expression study on IA by Pera *et al.* found this gene to be differentially expressed in the Polish population [48]. Although the PTPRB gene is not found to be differentially expressed in the JP population, PANOGA was able to identify this gene as important for the IA development mechanism using GWAS data in the EU and JP populations.

## Discussion

The pathway and network oriented analysis of GWAS data in two different populations together with gene expression data gave us the tools to investigate the pathogenesis of IA. The genes that are found to be targeted by disease predisposing SNPs are shown to be involved in several biological pathways including MAPK signaling, Cell cycle, TGF-beta signaling, Focal adhesion, Adherens junction, Regulation of actin cytoskeleton, and Neurotrophin signaling pathways. Since these pathways are known to have a role in the regulation of cell growth, tissue remodeling, inflammation, and wound healing, they are likely to contribute to the pathophysiology of IA. In addition to these top ten pathways, here, we also would like to discuss in detail the identified signaling pathways from the top 20 list that are functionally relevant to the pathogenesis of IA.

**Figure 1:**
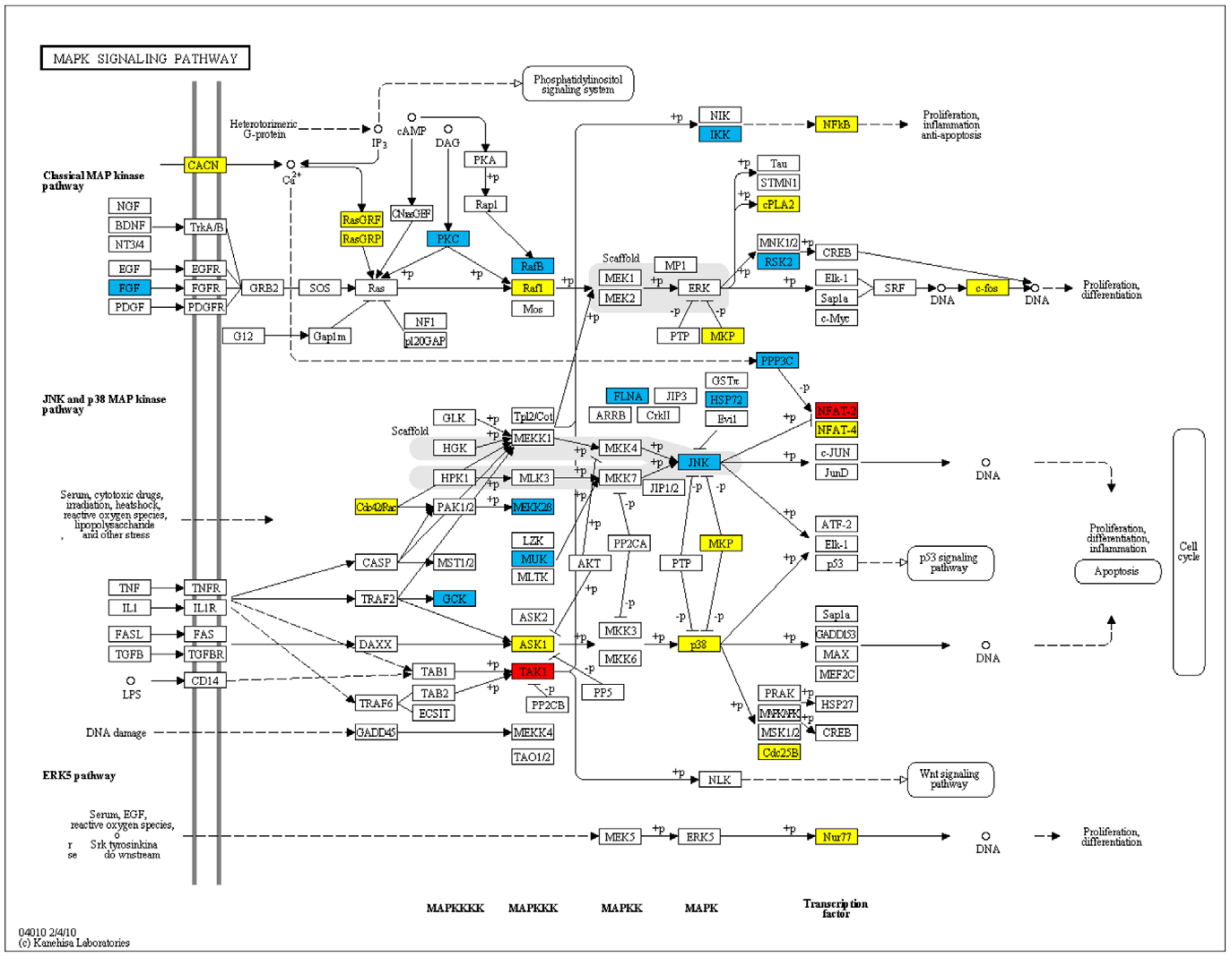
KEGG pathway for MAPK signaling. The set of genes shown in blue includes genes that are found by EU GWAS; yellow includes genes that are found by JP GWAS; red includes genes that are found both by EU and JP GWAS.

The mitogen-activated protein kinases (MAPKs) are serine-threonine kinases that are involved in intracellular signaling related with several cellular activities such as cell proliferation, differentiation, survival, death and transformation [49], [50]. Laaksamo *et al.* studied the expression and phosphorylation of the 3 major MAPKs in unruptured and ruptured human IAs: c-Jun N-terminal kinase (JNK), p38, and extracellular signal-regulated kinase [51]. Their study shows that JNK and p38 expression have a role in IA growth; and JNK activity and expression have possible roles in rupture [51]. As shown in Table 1, this pathway is identified at 1st and 8th rankings with P = 3.53E−27, P = 2.70E−18 in EU and JP populations, respectively. As shown in Figure 1 in red, and in Table S4, in this pathway, MAP3K7 (TAK1) and NFATC2 genes are identified in our method both by EU and JP GWAS. There are 28 typed SNPs on the MAP3K7 gene according to EU GWAS and 2 typed SNPs according to JP GWAS; and among those SNPs, only 1 SNP is identified in both studies. As shown in the KEGG pathway map in Figure 1, the TAK1 gene is shown to have a downstream effect on Wnt signaling and the pathways of proliferation, inflamation, and anti-apoptosis. Additionally, as part of this pathway, HSPA1L, PRKCA, BRAF, RPS6KA2, MAP3K2, MAP4K2, PPP3CA, MAPK10, FGF12, FLNB, CHUK, MAP3K12 genes are uniquely found in EU population (shown in blue in Figure 1) and DUSP10, RAF1, NR4A1, NFKB1, CACNG2, CDC25B, FOS, PLA2G4A, RPS6KA3, MAP3K5, RASGRP3, RASGRF1, MAPK14, RAC1, NFATC4, CACNA1C genes are uniquely found in JP population (shown in yellow in Figure 1).

Several putative risk genes were suspected to play a role in cell-cycle progression, potentially affecting the proliferation and senescence of progenitor-cell populations that are responsible for vascular formation and repair [39]. As shown in Table 1, Cell-cycle pathway is identified at 2nd and 4th rankings with P = 2.35E−25, P = 2.81E−19 in EU and JP populations, respectively.

**Figure 2:**
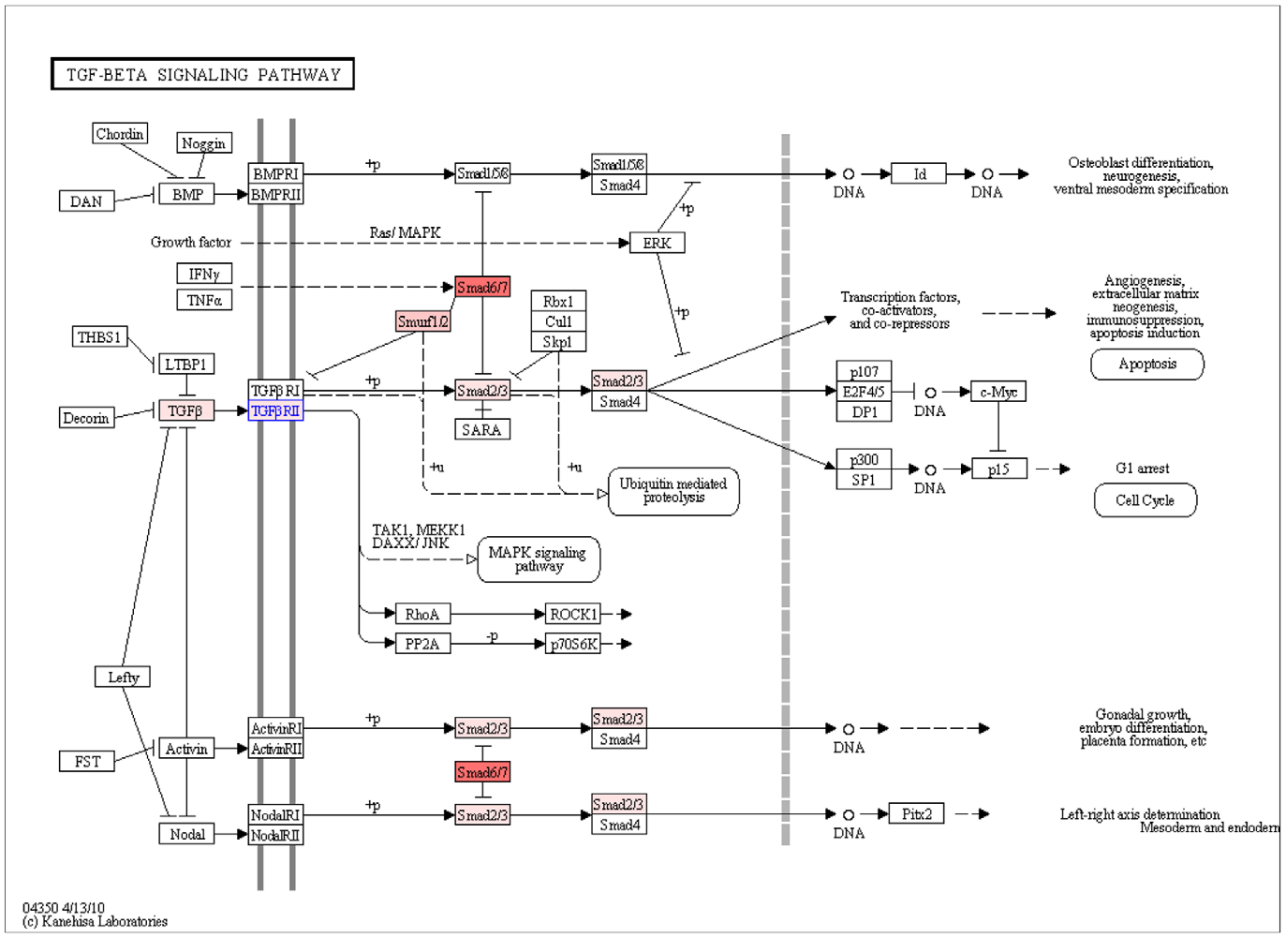
KEGG pathway map for TGF-beta signaling pathway. The shade of red color in genes indicates the number of targeted SNPs in JP population per base pair of the gene. Red refers to the highest targeted gene, whereas white refers to a gene product, not targeted by the SNPs. Blue border indicates that the gene is found to be differentially expressed.

The transforming growth factor-beta (TGF-beta) signaling pathway is known to play a role in aortic aneurysms and also has a possible role in aneurysms in general [52]. Additionally, TGF-beta signaling is shown to drive aneurysm progression in multiple disorders, including Marfan syndrome [53]. It is reported that the therapies that inhibit this signaling cascade are already in clinical trials in mice [53]. As shown in Table 1, this pathway is identified at 3rd and 9th rankings with P = 6.26E−24, P = 2.41E−17 in EU and JP populations, respectively. In our analysis, we detected 15 and 9 SNP targeted genes in EU and JP populations, respectively. As shown in Table S4, 5 of these genes (SMAD6, SMAD3, SMAD2, SMURF1, TGFB2) are identified in both populations; and 2 of these 5 genes, SMAD3 and SMAD6, have common typed SNPs. SMAD2 in this pathway harbors 28 typed SNPs in the EU population which is not observed in the JP population. In Figure 2, the KEGG pathway map of TGF-beta signaling shows that the SMAD6 gene (shown in red) is targeted by typed SNPs in JP population and it inhibits the formation of SMAD2/3 complex (shown in pink). The colors of the genes in Figure 2 indicate the number of targeted SNPs in JP population per base pair of the gene, from red to white. SMURF1 (shown in pink) inhibits TGFBR2 (shown in pink with blue border), which also binds to TGFB (shown in pink). The TGFBR2 gene is found to be differentially expressed. As a downstream effect, the SMAD2/3 complex (shown in pink) is affected as well as the transcription factors, co-activators, and co-repressors. As shown in Figure 2, this cascade of events leads to angiogenesis and neogenesis. Our method detected ten additional genes (ACVR2B, SMAD9, SMAD7, GDF5, SMAD4, SMAD1, BMP7, BMPR1B, BMPR1A, BMP6) that are affected in the EU population, but not in the JP population. These genes are not colored in Figure 2.

**Figure 3:**
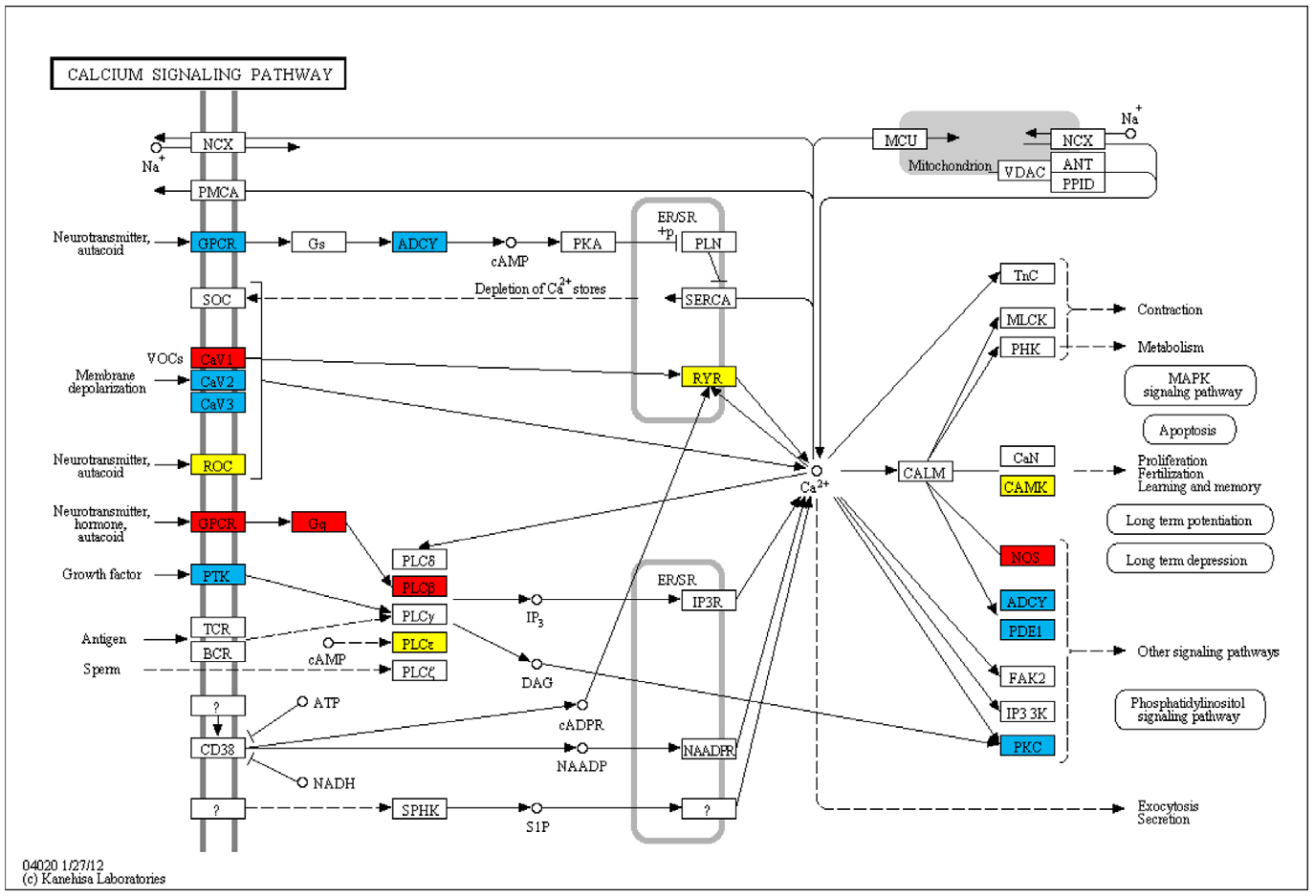
KEGG pathway for calcium signaling. The set of genes shown in blue includes genes that are found by EU GWAS; yellow includes genes that are found by JP GWAS; red includes genes that are found both by EU and JP GWAS.

Calcium is a key signaling ion that controls many different cellular processes, such as gene transcription, synaptic activity, muscle contraction, cell-cell communication, adhesion, and cell proliferation [54], [55]. The calcium signaling pathway has a significant role in regulating a great variety of neuronal processes [56]. As shown in Table 1, we identify this pathway in 20th and12th rankings with P = 3.27E−15, P = 1.37E−15 in EU and JP populations, respectively. In this pathway, we suspect a mechanism related to autocoids and GPCRs for IA disease development. As shown in Table S4 and in red in Figure 3, GPCR, Gq, PLCB1 genes are detected in our methodology both by EU and JP GWAS. These genes are found on our suspected autocoid path in calcium signaling pathway. There are 44 marker SNPs on PLCB1 gene according to EU GWAS and 1 marker SNP according to JP GWAS; furthermore, none of those SNPs are identified in both studies. As part of our suspected autocoid path, Kuo *et al.* has shown the association of a polymorphism of ITPKC (*inositol-trisphosphate 3-kinase C, IP3-3KC*) with the susceptibility and aneurysm formation in KD patients in a Taiwanese population [57]. ITPR1 *(inositol 1.4.5-trisphosphate receptor, type 1, IP3R)* is identified in our analysis as part of a Calcium signaling pathway and it is also found as differentially expressed between aneurysm patients and controls in JP population. The calcium signaling pathway’s high rank in our analysis and our suspected autocoid path within this pathway also corroborate recent reports that Clazosentan is in a phase III trial to reduce vasospasm caused by Endothelin A autocoid [58], [59].

In this article, we have described the advantages of a network and pathway-oriented analysis of GWAS data on different populations. Starting with two independent GWAS, which are conducted on two different populations, we have shown that most of the affected pathways are shared between populations. But, in different populations, different SNP targeted genes are found to be affected in these commonly found pathways. In other words, same pathways can be targeted via independent genes in different populations. Even though there are not so many common disease predisposing SNPs and commonly targeted genes between two populations, the identification of 7 common pathways in the top 10 pathways showed the relevance of our pathway-oriented approach.

As explained in the Introduction section of the manuscript, one advantage of studying different populations in GWAS is that the risk variants can vary in their occurrence across populations [23], [24]. For example, while the high-risk variant at MYBPC3 gene is observed with a frequency of ∼4% in cardiomyopathy patients in Indian populations; this variant is rare or absent in other populations [25]. Another reason is the difference in allele frequencies and biological adaptations among populations, which in turn affects the detectability and importance of risk variants. The identification of a variant might be easier in some populations when compared to that of other populations since the particular histories of recombinations, mutations, and divergences of genealogical lineages in the various populations affect the mappability of a variant. This situation is observed in the variants of TCF7L2 and KCNQ1 genes in type 2 diabetes [26], [27]. Additionally, environmental factors, diet, or population specific genetic differences might also have a role on the disease causing affected pathways specific to a population.

It should be kept in mind that pathway-based analyses, like the one presented here, are limited to our knowledge of cellular processes. The biological functions of most of the genes in the genome are unknown. Since network and pathway tools make use of functional information from gene and protein databases, they are biased toward the well-studied genes, interactions, and pathways. Also, this study only evaluates the variants associated with genes represented in the protein-protein interaction network. Nevertheless, there is a scope for the development of related methodologies to increase the power to detect associations in these genes. As shown in this paper, attempts maybe initiated to overcome such limitations via combining information from several sources (functional properties of SNPs, PPI network) and the genetic association of a SNP with the disease.

In the past, drug development was limited to a few hundred targets, which were deeply understood. As a result of advances in molecular technologies, thousands of new potential drug targets have been discovered, but their mechanisms of action and potential “druggability” are as of yet not well understood. GWAS are one such example of these advances in molecular technologies that uncovers well-validated genetic risk factors for common diseases [60], [61]. Several known drug targets are identified in GWAS; and it is estimated that previously unknown targets are buried in the treasure of GWAS data [12]. In addition to the hidden drug targets, GWAS analyses are thought to provide several potential opportunities for clinical intervention [12]. With rapid technological developments and continuous data production in the field of GWAS, more and more datasets are expected to be available in the near future. To pay off the huge investments in GWAS, new strategies are expected to be developed [11]. The analysis of epistatic relationships of the identified variants in GWAS or the assessment of combined risk for groups of functionally associated genes (defined by PPI networks or pathways) is advocated to significantly increase the amount of common complex disease risk information that can be extracted from GWAS data sets [11], [62]. In this respect, we propose a novel method for the identification of pathway markers using GWAS data in different populations. By applying our method on the IA dataset, we have shown that while the shared pathways between the EU and the JP populations explain the general mechanisms of IA disease development, the pathways that are identified by population specific GWAS also need to be examined to gain a more comprehensive understanding of IA pathogenesis. Each population may search for disease causing factors targeting the genes within these affected pathways. Population specific function altering modifications within these pathways can be used as markers for early diagnostics as part of preventive health care. Rather than the population, the same method can be extended to individuals to identify modifications occurring on the genes within the marker pathways. Hence, we can determine individual causes of disease development that may be exploited for drug development and personalized therapeutic applications. More importantly, our method also gives the functional effect of the SNP’s on the targeted genes in marker pathways; furthermore, this information can be exploited for individual therapy applications rectifying the impact of these function-altering factors.

## Materials and Methods

### Genetic Association Data of Intracranial Aneurysm

Two intracranial aneurysm (IA) genome wide association study (GWAS) datasets have been used in this study. The first GWAS is a multicenter collaboration in Finnish, Dutch and Japanese cohorts totaling 5891 cases and 14,181 controls [39]. This study tested ∼832,000 genotyped and imputed SNPs using the Illumina platform. In personal communication with the authors, upon our request, JP population specific data was removed and EU population specific results were obtained, including 2780 cases and 12,515 controls. The second GWAS tested 312,712 SNPs on 1069 Japanese IA patients and 904 Japanese controls using the HumanHap300 or HumanHap300-Duo Genotyping BeadChips (Illumina) [40]. For both datasets, SNP data and the genotypic p-values of association for each tested SNP (calculated via Cochran-Armitage trend test) were obtained from our collaborators.

### Protein-protein Interaction (PPI) Data

A human protein-protein interaction (PPI) data-set was obtained from the supplementary material of [63]. This dataset is composed of two high quality systematic yeast two-hybrid experiments [64], [65] and PPIs obtained from literature by manual curation [64]. The integrated set of PPIs contains 61,070 interactions between 10,174 genes.

### Gene Expression Dataset

A list of differentially expressed genes along with their p-values was obtained from the study of Krischek *et al.* [47]. In this study, four unruptured and six ruptured IA specimens, collected during 42 months, were used as cases. Four arteriovenous malformation (AVM) feeders, which were obtained during microsurgical resection, were used as intracranial control tissue. The average age of the IA patients was 56.4 years, and that of the controls was 60.25 years. All patients and controls were of Japanese ethnicity. All tissue samples were profiled using oligonucleotide microarrays (Agilent Technologies). In the original study, in order to find out the differentially expressed genes between the aneurysmal cases and the controls, the analytical tools in the GeneSpringGX v11 was utilized. The statistical significance of the difference between the gene expression levels was calculated via the Student’s t-test [47]. In our study, we used those genes showing significant difference at the false discovery rate of 0.05 according to the Benjamini and Hochberg procedure [66].

### Network and Pathway-Oriented GWAS Analysis

**Figure 4:**
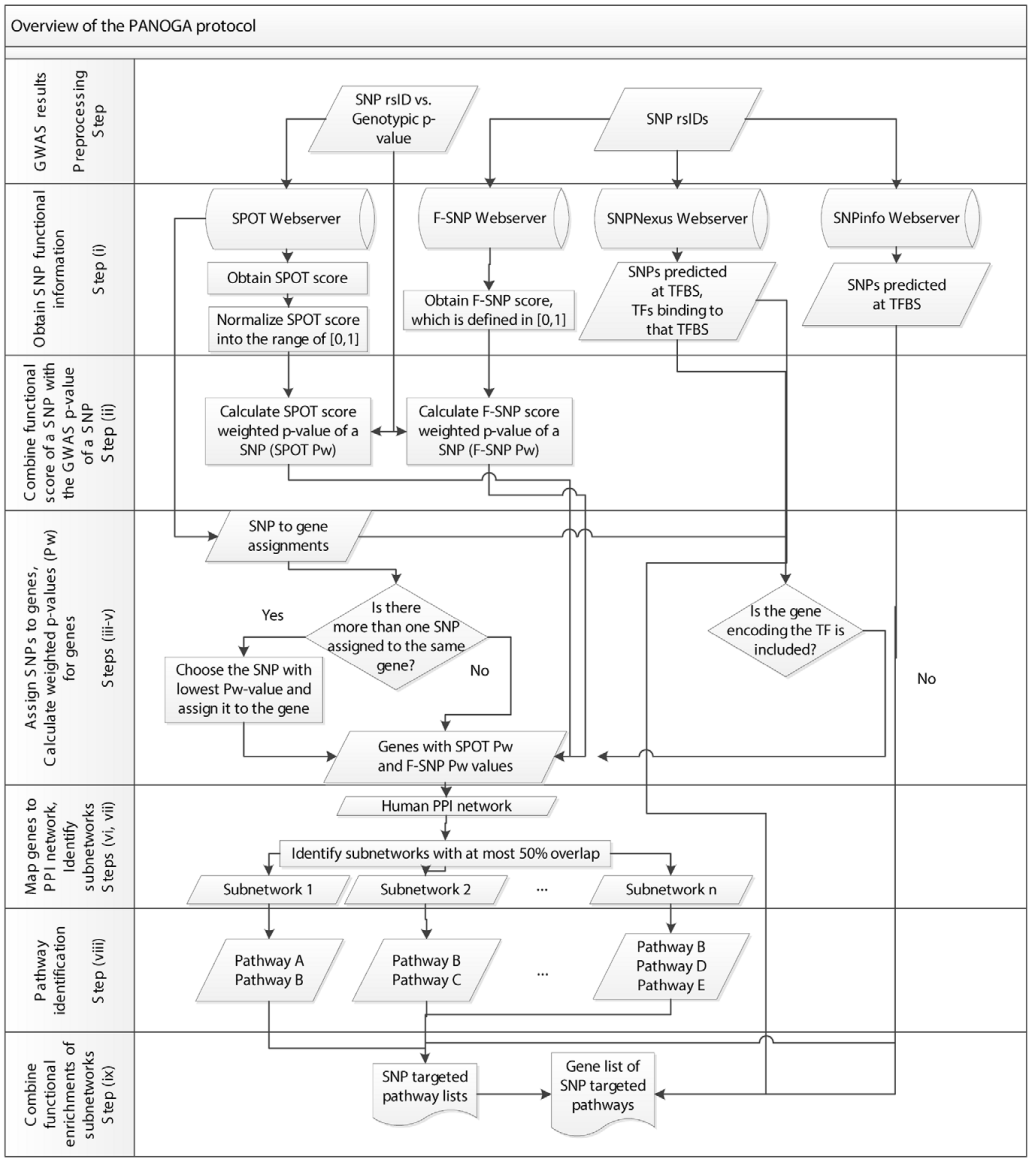
Outline of our assessment process. In steps (i) to (v), a gene-wise Pw-value for association with disease was computed by integrating functional information. In step (vi), Pw-values were loaded as two separate attributes of the genes in a PPI network. In step (vii), active sub-networks of interacting gene products that were also associated with the disease, are identified. In step (viii), genes in an identified active sub-network were tested whether they are part of functionally important KEGG pathways. Lastly, step (ix) integrates the functional enrichments of the generated sub-networks.

Starting with a list of SNPs associated with disease in GWAS, PANOGA identifies SNP targeted KEGG pathways. In our study, GWAS results are used in the form of SNP rs ids vs. p-values, where the p-values refer to the genotypic p-values of association for each tested SNP. We only focused on SNPs with nominal evidence of association (P<0.05) in a GWAS, following the study in [14]. PANOGA proceeds in nine steps, as outlined in Figure 4. In most post-GWAS analyses, computing gene-wise P values is one of the key steps. In this respect, our methodology firstly calculates SNP-wise statistics, secondly it assigns SNPs to genes, and finally it calculates gene-wise statistics, as shown in the steps (i) to (v) of PANOGA protocol in Figure 4.

#### Calculation of SNP-wise statistics (Steps (i) and (ii))

Inspired by the Saccone et al’s study [67], the SNP-wise statistics used in our methodology combines the functional scores of SNPs with genotypic p-values. As it is well known, SNPs might have different functional impacts such as: an effect on transcriptional regulation by changing TFBS’s activity; premature termination of amino-acid sequence (generate a stop codon); alteration in the splicing pattern or efficiency by disturbing splice site, exonic splicing enhancers (ESE) or silencers (ESS); a change in protein structures or properties by altering single amino acids or changing the frame of the protein-coding region; regulation of protein translation by affecting microRNA (miRNA) binding sites activity. Among the existing SNP functional effect prediction tools, step (i) of PANOGA utilizes SPOT [67] and F-SNP [68] web-servers. SPOT score [67] takes into account SNP/gene transcript functional properties (including nonsense, frameshift, missense and 5′ and 3′-UTR designations), impact of an amino acid substitution on the properties of the protein product from PolyPhen server [69], [70], evolutionary conserved regions from ECRbase [71], and all possible LD proxies - SNPs with r^2^ over a predefined threshold in a specific HapMap sample [72]. On the other hand, F-SNP score incorporates: functional effects of SNPs, predicted at the splicing, transcriptional, translational, and post- translational levels [68]. F-SNP Score is defined in the range of [0,1], where 0 means the functional consequence of a SNP on the gene product is negligible and 1 means the functional consequence of the SNP on the gene product is serious [73]. SPOT scores are not limited to a range of [0,1]; hence, we normalized SPOT scores to this range. To be able to calculate SNP-wise statistics, we followed Saccone et al’s formulation [67] in step (ii) as following. In this step, PANOGA combines the functional scores (FS) obtained from SPOT and F-SNP web-servers with GWAS p-values (P/10̂FS) [67] and calculates a weighted p-value, Pw, for each score, for each SNP [21].

#### Assigning SNPs to genes (Step (iii))

In step (iii), SNPs are assigned to genes using SPOT’s SNP to gene assignment module [67]. SPOT considers all known SNP/gene transcript associations and their functional impacts; and then it assigns the SNP to the gene with the highest priority [67]. To generate those SNP/gene transcript associations, SPOT program utilizes information from the PolyPhen method of predicting the effect of an amino acid substitution on the properties of the protein product [69], [70]. Those effects can be directly detected from DNA and RNA sequences, like nonsense and missense amino acid substitutions, untranslated regions, coding regions, and frameshifts. SPOT [67] also takes into account evolutionary conserved regions from ECRbase [71], and all possible LD proxies - SNPs with r^2^ over a predefined threshold in a specific HapMap sample [72]. Hence, by prioritizing all known SNP/gene transcript consequences, propitious association signals found in GWAS are not lost at the SNP to gene transition step.

#### Calculation of gene-wise statistics (Steps (iv) and (v))

Once the SNPs are assigned to genes, one needs to calculate gene-wise statistics using the attributes of those SNPs. In step (iv), SPOT [67] and F-SNP [68] Pw-values are assigned to each gene as two separate attributes. If more than one SNP is assigned to the same gene, the most widely applied method in the field is to select the SNP with the smallest P value among all SNPs mapped to a gene [74]. Following this tradition, in such cases, SPOT and F-SNP Pw values of all these SNPs are taken into account and lowest SPOT and F-SNP Pw values are assigned to the gene. In step (v), a possible overlap of the input SNPs with known Transcription Factor Binding Sites (TFBS) at TRANSFAC [75] is also checked. If this TF is not already found in step (iii), this TF is added to our list by transferring its SPOT and F-SNP Pw-values from its associated SNP.

#### Sub-network Identification (Steps (vi) and (vii))

In step (vi), genes with two separate weighted P-values (Pw values) are mapped to a human protein protein interaction network. By using the Pw values of the genes and network topology, step (vii) aims to find out active sub-networks in the human PPI network using jActive Modules algorithm [76]. Although this algorithm was originally developed for microarray gene expression data, steps (i)-(v) of PANOGA successfully adapts GWAS data to be used with this algorithm. In terms of GWAS data, the jActive Modules algorithm integrates the network topology with the calculated Pw-values of each gene to extract potentially meaningful active sub-networks. Here, an active sub-network refers to a connected subgraph of the interactome that has high total significance of genotypic p-values of the disease-predisposing SNPs with respect to the controls. It should be noted that in this algorithm, an identified sub-network with a high score is not necessarily the sub-network that includes the genes with very significant genotypic p-values. Instead, the identified sub-network can be composed of many genes with moderately significant genotypic p-values. Hence, this algorithm helps to discover groups of genes that display seemingly negligible association with aneurysm when evaluated individually but when considered as a group, display strong association.

Based on the aggregate degree of genetic association with IA (S score), we identified 482 significant sub-networks for EU population specific dataset and 376 significant sub-networks for JP population specific dataset. In this analysis, only the sub-networks with S>3 were reported as significant, as stated in the original publication [76]. Previously, we focused only on the highest scoring sub-network. However, we noticed that the scores of the identified sub-networks were very close to one other. We also realized that the highest scoring sub-network does not cover the initial PPI network; thus, we loose information. In the improved PANOGA, our response to this challenge is to combine pathway enrichment results of the identified sub-networks. At this stage, due to the nature of the search algorithm, several of these sub-networks overlap extensively in their component genes. Overlap threshold parameter defines the max level of identity between the constituent genes of any two identified sub-networks, in terms of percent. e.g. Overlap threshold parameter 0.8 indicates max 80% of the genes from one sub-network can be identical to the genes of another sub-network. When this parameter is set as too high, identical genes appear in any two different sub-networks. When this parameter is set as too low, moves to the different parts of the network is not abled. In PANOGA, with the generated sub-networks, we wanted to cover the whole human protein protein interaction network as much as possible. But at the same time, we did not want to include the same genes over and over in our sub-networks. To this end, starting with 0, we experimented PANOGA with 0.1 increments of the overlap threshold values. In this experiment, we compared the coverage of the PPI network. The coverage of the PPI network was 20%, 22%, 37%, 46%, 39%, and 32% for overlap threshold values 0, 0.2, 0.4, 0.5, 0.6, 0.8, respectively. As a result of this experiment, we decided to set the overlap threshold parameter as 0.5, which resulted in the maximum coverage. So, in PANOGA, rather than focusing on the highest scoring sub-network, we found all significant sub-networks that overlap less than 50% with each other.

#### Pathway Identification (Steps (viii) and (ix))

Following the identification of sub-networks, we evaluated whether these sub-networks were biologically meaningful in step (viii) of PANOGA. For each sub-network, we computed the proportion of the genes in an identified sub-network that are also found in a specific human biochemical pathway, compared to the overall proportion of genes described for that pathway. KEGG pathways that might have a role in IA disease mechanism are identified separately for the EU population and JP population by including the pathway in our final list, if it is found as significant for at least one of the identified sub-networks. We used two-sided (Enrichment/Depletion) test based on the hypergeometric distribution to examine the association between the genes targeted by IA predisposing SNPs and the genes in each KEGG pathway. To correct the P-values for multiple testing, the Bonferroni correction procedure is applied. In this pathway identification step, we used the command-line version of ClueGO_v1.4 on hundreds of the identified sub-networks [77]. While an identified sub-network represents only one part of the whole interaction network, the identified pathways for this sub-network represent one aspect of the disease. Since the human complex diseases are multifactorial, by discovering the pathways from different sub-networks, we aimed to enlighten different aspects of the disease. To this end, step (ix) integrates the functional enrichments of the generated sub-networks. If a KEGG pathway is found to be statistically significant for at least one of the active sub-networks with S score >3, PANOGA adds this pathway as associated with disease into our final list of significant KEGG pathways. For each identified KEGG pathway in our final list, PANOGA also counts the number of associated SNPs from GWAS, the number of regulatory SNPs among those disease predisposing SNPs (SNPs located on TFBSs or miRNAs), the number of SNP-targeted genes, the number of sub-networks that this pathway is found to be statistically significant [45]. PANOGA is applied separately to the GWAS of IA on EU and JP populations.

### Network and Pathway-Oriented Microarray Data Analysis

First of all, 1418 differentially expressed genes are mapped into PPI network and their p-values are used as gene attributes. Secondly, sub-network identification is conducted using jActive Module [76]. Lastly, in the functional enrichment step, genes are assigned into functionally relevant KEGG pathways by combining a network-oriented approach with pathway-oriented approach, as developed in [21].

## Supporting Information

Table S1 **The whole list of the identified KEGG pathways, which are found in European population as important for IA development mechanism.**
(XLS)

Table S2 **The whole list of the identified KEGG pathways, which are found in Japanese population as important for IA development mechanism.**
(XLS)

Table S3 **The whole list of the identified KEGG pathways, which are found in both European and Japanese populations as important for IA development mechanism.**
(XLS)

Table S4 **The top 20 over-represented KEGG pathways.** Seven out of the top ten affected pathways in both EU and JP populations are shown in italic. SNP Targeted Genes that are identified in both EU and JP populations are shown in the last column; along with the number of commonly typed SNPs in both populations, only in EU population and only in JP populations are shown in paranthesis.
(DOC)

Table S5 **The top 20 over-represented KEGG pathways, which are identified for IA gene expression dataset.**
(DOC)
