# Supplementary material for: The Identification of Pathway Markers in Intracranial Aneurysm Using Genome-Wide Association Data from Two Different Populations
Source: PLoS One. 2013 Mar 6;8(3):e57022. doi: 10.1371/journal.pone.0057022 (PMC3590201; doi:10.1371/journal.pone.0057022)
Supplement: Table S4 — The top 20 over-represented KEGG pathways. Seven out of the top ten affected pathways in both EU and JP populations are shown in italic. SNP Targeted Genes that are identified in both EU and JP populations are shown in the last column; along with the number of commonly typed SNPs in both populations, only in EU population and only in JP populations are shown in paranthesis. (DOC) [file pone.0057022.s004.doc]

| **KEGG Term** | **Any Common SNPs in Common Genes?** | **Commonly Associated, SNP Targeted Genes and their SNP Counts: (Common) (EU GWAS) (JP GWAS)** |
| --- | --- | --- |
| *MAPK signaling pathway* | Y | MAP3K7 (1)(28)(2), NFATC2 (0)(1)(2), |
| *Cell cycle* | Y | SMAD3 (1)(14)(4), SMAD2 (0)(28)(1), |
| *TGF-beta signaling pathway* | Y | SMAD6 (2)(7)(4), SMAD3 (1)(14)(4), SMAD2 (0)(28)(1), SMURF1 (0)(4)(3), TGFB2 (0)(6)(1), |
| ErbB signaling pathway | N |  |
| *Focal adhesion* | Y | IGF1R (0)(2)(5), LAMA1 (0)(1)(6), ITGB6 (0)(4)(4), ITGA1 (0)(5)(2), ITGB5 (1)(5)(3), |
| Proteasome | N |  |
| *Adherens junction* | Y | PTPRB (1)(2)(1), PTPRM (0)(10)(6), |
| Notch signaling pathway | N | NCOR2 (0)(2)(1), |
| *Regulation of actin cytoskeleton* | Y | ITGB5 (1)(5)(3), |
| *Neurotrophin signaling pathway* | N | RPS6KA2 (0)(6)(1), |
| Chronic myeloid leukemia | Y | SMAD3 (1)(14)(4), |
| Apoptosis | N | BID (0)(1)(1), |
| Pathways in cancer | Y | SMAD3 (1)(14)(4), CSF1R (0)(3)(1), |
| Tight junction | Y | MAGI2 (0)(12)(11), EPB41 (2)(8)(5), MPDZ (0)(4)(1), PRKCE (0)(4)(7), JAM3 (1)(1)(1), CTNNA2 (1)(12)(3), |
| Long-term potentiation | N | GRIN2A (0)(7)(1), PLCB1 (0)(44)(1), GRM1 (0)(1)(3), |
| Measles | N |  |
| T cell receptor signaling pathway | N |  |
| Nucleotide excision repair | N | RPA3 (0)(1)(2), |
| Chemokine signaling pathway | N | VAV3 (0)(8)(1), PLCB1 (0)(44)(1), |
| Calcium signaling pathway | Y | GNA14 (0)(5)(1), NOS1 (0)(1)(1), ADCY2 (0)(8)(5), CHRM2 (0)(1)(1), ADRA1A (0)(2)(4), PLCB1 (0)(44)(1), CACNA1C (1)(3)(5), GRM1 (0)(1)(3), |
