# Supplementary material for: The Identification of Pathway Markers in Intracranial Aneurysm Using Genome-Wide Association Data from Two Different Populations
Source: PLoS One. 2013 Mar 6;8(3):e57022. doi: 10.1371/journal.pone.0057022 (PMC3590201; doi:10.1371/journal.pone.0057022)
Supplement: Table S5 — The top 20 over-represented KEGG pathways, which are identified for IA gene expression dataset. (DOC) [file pone.0057022.s005.doc]

|  | **KEGG Term P-values Corrected with Bonferroni** | | | **Rankings** | | |
| --- | --- | --- | --- | --- | --- | --- |
| **KEGG Term** | **Gene Expression** | **GWAS EU** | **GWAS JP** | **Gene Expression** | **GWAS EU** | **GWAS JP** |
| Ribosome | 7.91E-23 | 1.40E-08 | 5.93E-19 | 1 | 73 | 5 |
| Spliceosome | 7.40E-17 | 2.05E-13 | 4.72E-13 | 2 | 33 | 27 |
| RNA transport | 3.97E-14 | 6.26E-09 | - | 3 | 69 | - |
| Complement and coagulation cascades | 6.05E-13 | 7.00E-14 | 1.06E-09 | 4 | 31 | 48 |
| T cell receptor signaling pathway | 7.86E-12 | 1.62E-16 | 1.97E-15 | 5 | 17 | 15 |
| ErbB signaling pathway | 5.70E-09 | 9.52E-22 | 2.47E-15 | 6 | 4 | 16 |
| Chronic myeloid leukemia | 6.70E-09 | 2.62E-18 | 8.13E-11 | 7 | 11 | 36 |
| Natural killer cell mediated cytotoxicity | 9.96E-09 | 2.56E-07 | 1.29E-09 | 8 | 81 | 50 |
| RNA degradation | 1.44E-08 | 3.44E-11 | 1.66E-07 | 9 | 44 | 67 |
| Osteoclast differentiation | 1.45E-08 | 8.12E-15 | 4.97E-10 | 10 | 26 | 43 |
| Neurotrophin signaling pathway | 6.68E-08 | 2.49E-18 | 1.92E-18 | 11 | 10 | 7 |
| Adherens junction | 1.74E-07 | 4.91E-19 | 2.58E-21 | 12 | 7 | 1 |
| mRNA surveillance pathway | 3.59E-07 | - | - | 13 | - | - |
| Pyruvate metabolism | 1.87E-06 | - | 5.82E-05 | 14 | - | 92 |
| Toll-like receptor signaling pathway | 3.26E-06 | 9.18E-13 | 1.50E-10 | 15 | 35 | 38 |
| Small cell lung cancer | 3.55E-06 | - | 1.01E-08 | 16 | - | 55 |
| Proteasome | 4.19E-06 | 2.35E-21 | 4.54E-11 | 17 | 6 | 35 |
| Focal adhesion | 8.57E-06 | 9.55E-22 | 5.60E-21 | 18 | 5 | 2 |
| Fc gamma R-mediated phagocytosis | 1.47E-05 | 4.00E-09 | 1.32E-13 | 19 | 66 | 22 |
| Toxoplasmosis | 2.68E-05 | 1.06E-08 | - | 20 | 72 | - |
